# Retrospective analysis of the outcomes of pulpotomies in traumatised permanent anterior teeth

**DOI:** 10.1111/edt.12781

**Published:** 2022-08-16

**Authors:** Lucy Yu, Bill Kahler, Shanika Nanayakkara, Neeta Prabhu

**Affiliations:** ^1^ School of Dentistry, Faculty of Medicine and Health The University of Sydney Sydney New South Wales Australia; ^2^ Department of Paediatric Dentistry Westmead Centre for Oral Health Westmead New South Wales Australia; ^3^ The University of Queensland Oral Health Centre Herston Queensland Australia

**Keywords:** complicated crown fractures, dental trauma, permanent teeth, pulp prognosis, pulpotomy

## Abstract

**Background/Aim:**

Complicated crown fractures are frequently encountered in the paediatric population and pulpotomy procedures (either partial or coronal) are recommended to maintain the pulp. The aim of this study was to determine the pulp outcomes of permanent teeth with complicated crown fractures treated with pulpotomy in a hospital‐setting and to identify potential factors which may influence the outcomes.

**Material and Methods:**

Data for this retrospective study were extracted from dental records of patients with complicated crown fractures and treated with pulpotomies at a single centre between 1 January 2015 and 30 August 2019. Pulp outcomes were determined, and the associations between the outcome and independent variables were assessed using the Chi‐Square test of independence and the Point‐Biserial Correlation Test. Predictors of outcome were identified using the binary logistic regression model.

**Results:**

The overall success of pulpotomy in managing traumatised permanent teeth was 61%, which was lower than those previously reported. Pulp healing was seen in 54.1% and 73.7% of teeth treated with partial pulpotomies and coronal pulpotomies, respectively. The presence of a radiographically detectable dentine bridge (*p* < .01) and longer clinical experience of the clinician (*p* < .04) was significantly associated with successful outcomes. The history of pain and the stage of root development were identified as significant predictors of the outcome.

**Conclusion:**

Pulpotomy is a viable treatment modality for complicated crown fractures in the paediatric population. However, appropriate case selection and further training may be required to ensure improved pulp healing outcomes. A longer follow‐up period should be considered to identify late‐stage complications.

## 1 INTRODUCTION

Traumatic dental injuries (TDIs) are frequently encountered amongst the paediatric population and often have long‐term consequences.^1^ Injuries that result in pulp exposure to the oral environment are described as complicated and they are classified as (1) complicated crown fractures (CCF) or (2) complicated crown‐root fractures (CCRF). Some authors do not consider CCRF as a separate entity and classify them as either crown fractures or root fractures.^2^ Irrespective of the type of fracture, the sequelae following traumatic pulp exposures are identical and preservation of the pulp should be considered as a treatment priority.

Conservative pulp therapies aim to maintain the remaining pulp and allow continued root development and apex formation in immature teeth.^1^ A pulpotomy is a procedure that involves the removal of the inflamed portion of the pulp, thereby allowing the remaining pulp to survive and maintain normal function. The aim of a partial pulpotomy, (also known as the Cvek pulpotomy), is to preserve the cell‐rich coronal pulp tissue which has a higher potential to promote healing compared with the radicular pulp.^3^, ^4^ Due to its high reported success rates between 86% and 100%, a partial pulpotomy is considered to be the treatment of choice for traumatic pulp exposures.^5^, ^6^, ^7^, ^8^, ^9^, ^10^, ^11^, ^12^, ^13^ When necrotic tissue or tissue with obviously impaired vascularity is present at the exposure site of an immature tooth, the pulpotomy should be performed at a deeper level where healthy fresh bleeding can be achieved.^2^ A coronal, or cervical, pulpotomy involves the complete removal of the coronal pulp tissue and the placement of a wound dressing at the root canal orifice.^4^ The reported success rates of coronal pulpotomies range between 72% and 90.2%.^9^, ^14^

In the recently updated trauma guidelines published by the International Association of Dental Traumatology (IADT), there has been a shift in preference to conservative pulp treatment, and partial pulpotomies are now the recommended treatment for teeth with both incomplete and complete root development.^1^ Compliance with the previous IADT guidelines resulted in a 95.7% survival rate for teeth with complicated crown fractures.^15^ The management of dental trauma is more time‐consuming and costlier than the majority of other outpatient accidental injuries.^16^ If the burdens of treatment from patient, time and financial perspectives are taken into consideration, it is essential to review the current understanding of the partial pulpotomy technique and the parameters for its success in order for clinicians to provide the best evidence‐based care to their patients.

The aims of this study were to (1) evaluate the outcomes of pulpotomy treatment on traumatised anterior permanent teeth with pulp exposures and (2) identify potential factors which may influence the outcomes after pulpotomy by retrospectively analysing clinical data.

## 2 MATERIALS AND METHODS

For this retrospective study, data were extracted from the dental records of patients treated at the Department of Paediatric Dentistry and Orthodontics, Westmead Centre for Oral Health (WCOH), Sydney, Australia, between 1 January, 2015 and 30 August 2019. These patients presented with one or more traumatised permanent anterior teeth requiring a pulpotomy. To be included in the study, the following criteria had to be fulfilled: (1) the traumatised teeth were diagnosed with a CCF or CCRF according to Andreasen's classification,^2^ (2) the traumatised teeth were treated with either partial or coronal pulpotomy and, (3) the follow‐up period after treatment was a minimum of 9 months with at least two appointments with accompanying radiographs. Teeth of all stages of root development were included. The data obtained from the records included: patient details, dental history, trauma history, details of the initial examination at WCOH, the treatment provided and follow‐up details. Ethics approval for the study was obtained through the Western Sydney Local Health District (WSLHD) Human Research Ethics Committee (HREC reference: 2009‐1 QA).

Complicated crown fractures were generally managed with either partial or coronal pulpotomies. While standardised protocols should ideally be followed, this does not always occur in real‐time clinical scenarios. The choice of treatment was dictated by the extent of the fracture, the amount of pulp removal required to establish adequate haemostasis and the operator's clinical judgement. In all cases, the Cvek protocol was followed.^7^ A minor modification was the use of a non‐setting calcium hydroxide (Ca[OH]_2_) (UltraCal® XS, Ultradent) as the pulp dressing material, followed by a layer of setting Ca(OH)_2_ (Dycal®, Dentsply). A resin‐modified glass ionomer cement liner was used in some instances prior to the placement of a restoration. When possible, the fractured tooth fragments were rebonded to the tooth. Otherwise, strip crowns were frequently used for resin composite (RC) restorations. Most procedures were performed under a dental dam.

The clinical follow‐up intervals were not standardised. At the follow‐up appointments, any reported patient symptoms, the response to pulp sensibility testing using cold spray (Roeko Endo‐Frost, Coltène), mobility, tenderness to percussion, discolouration and radiographic findings were recorded. Healing was considered to have taken place if the tooth was (i) asymptomatic, (ii) had no radiographically demonstrable intra‐radicular or peri‐radicular pathological changes and (iii) had continued root development if the tooth was immature.

The diagnosis of pulp necrosis (PN) was made when at least two of the following three criteria were fulfilled (1) pain, (2) tenderness to percussion, and (3) a periapical radiolucency. The stage of root development was categorised based on Cvek's classification.^17^ Two researchers independently reviewed the radiographs. Where there was disagreement, the radiographs were reviewed together to reach agreement.

The data collected were entered manually into an MS Excel spreadsheet (Microsoft Office 365 Excel). Statistical analysis of extracted quantitative data from the dental records was performed using IBM SPSS Statistics (version 26, IBM SPSS Inc.). Descriptive statistics were reported as frequencies and proportions for categorical variables and mean (±SD) for continuous variables. The association between variables was assessed using the Chi‐Square test of independence and the Point‐Biserial Correlation test. Following bivariate analysis, variables with a *p*‐value < .05 were entered into a binary logistic regression model to identify the predictors of the outcome. Age of the patient, root development stage, time interval from injury and pain during the follow‐up were incorporated into the model. The Hosmer–Lemeshow goodness of fit test resulted a *p*‐value > .05 for the model used. For all analyses, *p* < .05 was considered statistically significant.

## 3 RESULTS

The study population consisted of 50 patients with a total of 60 traumatised teeth requiring pulpotomy treatment. Thirty‐three patients were males (66%) and 17 patients (34%) were females. Their ages ranged between 7 and 15 years with a mean age of 10.1 (±2.1) years. Injury was most frequent in the 8‐ and 10‐years‐old age groups (20% each), followed by the 9‐years‐old group (18%). Home/indoors (24%) were frequently identified as the location of the injury. Falls (34%) were the most common cause, followed by collisions (22%). Patients predominantly sought primary care at WCOH (64%). Other primary care centres included were the Children's Hospital Westmead (4%), emergency departments of other hospitals (6%), private dental practices (24%) and unknown (2%). The primary care centre was not recorded for one patient (2%). Forty percent of the patients had accompanying soft tissue injuries. Maxillary central incisors (86.7%) were the most frequently injured teeth. CCF (93.3%) accounted for most injuries requiring treatment. Concomitant injuries were identified in 30% of cases, and these included lateral luxation (*n* = 2/60), subluxation (*n* = 9/60), extrusion (*n* = 1/60), mid‐root fracture (*n* = 1/60) and concussion (*n* = 4/60). The demographics and details of the injuries according to the pulp status at follow‐up are summarised in Table 1. There was no significant association between age and pulp healing. Teeth that had sustained CCF had only a slightly improved healing outcome than those with CCRF (*p* = .65). In terms of root development, the highest percentage of pulp healing was observed in teeth with stage 3 root development (71.4%). The time interval between injury and provision of treatment was not significantly associated with pulp healing (*p* = .31).

**TABLE 1:** Demographics and details of the injury of the patients according to the pulp status at follow‐up

	Pulp healing *N* (%) or mean (±SD)	Pulp necrosis *N* (%) or mean (±SD)	*p*‐Value^a^	Odds ratio (95% CI)
Age (Years)	10.2 (±2.03)	9.64 (±1.96)	.35	‐
Concomitant injury (*n* = 13)	7 (53.8%)	6 (46.2%)	.63	0.79 (0.21–2.52)
Soft tissue injury (*n* = 23)	16 (69.6%)	7 (30.4%)	.26	1.91 (0.62–5.85)
Type of fracture				
CCF (*n* = 52)	32 (61.5%)	20 (38.5%)	.65	1.60 (0.21–12.28)
CCRF (*n* = 4)	2 (50%)	2 (50%)		
Stage of root development				
3 (*n* = 7)	5 (71.4%)	2 (28.6%)	.50	
4 (*n* = 18)	9 (50%)	9 (50%)		‐
5 (*n* = 31)	20 (64.5%)	11 (35.5%)		
Time interval (h)				
0–12 (*n* = 20)	13 (65.0%)	7 (35%)	.31	
12–24 (*n* = 18)	11 (61.1%)	7 (38.9%)		‐
24–48 (*n* = 5)	1 (20.0%)	4 (80.0%)		
>48 (*n* = 6)	3 (50%)	3 (50%)		

Abbreviations: CCF, complicated crown fracture; CCRF, complicated crown‐root fracture.

^a^ Pearson Chi‐square test.

The distribution of treatment variables according to the pulp status at follow‐up are summarised in Table 2. The outcome of partial pulpotomy was less favourable than for coronal pulpotomy with an OR of 0.420 for pulp healing (95% CI 0.125–1.407). Also, 73.7% of teeth treated with coronal pulpotomy demonstrated pulp healing, while only 54.1% of teeth treated partial pulpotomy had favourable healing outcomes. Post‐graduate registrars performed 50% of the treatment, while the remainder of cases were managed by staff specialists (23%) and general dental practitioners (GDP) (27%). The post‐graduate registrar training period was 3 years, but their previous GDP experience ranged between 2 and 10 years. Specialist experience within the department ranged between 1 and 20 years, and GDPs that were rotated through the department had 1–2 years of clinical experience. Treatment provided by post‐graduate and specialists in Paediatric Dentistry had better outcomes than those managed by general dental practitioners (GDP) (*p* = .040). Pulp healing was seen in 70.4% and 71.4% of teeth treated by post‐graduate registrars and specialists, respectively. Meanwhile, only 33.3% of teeth treated by GDPs were successful in this cohort. The type of restoration did not have a significant influence on pulp healing (*p* = .71). The use of strip crowns did not have a significant impact on the outcomes. Complications reported during the follow‐up period included lost restoration (46.7%), thermal sensitivity (33.3%), pain (31.7%), repeat traumatic injury (28.3%) and discolouration (21.7%). The distribution of post‐operative variables according to pulp status at follow‐up are summarised in Table 3. Pain at the follow‐up assessments was associated with significantly poorer outcomes (*p* = .001). Meanwhile, the presence of a dentine bridge at the site of the pulpotomy was associated with a significantly improved outcomes (OR 10.68, 95% CI 2.634–43.48, *p* = .001). In one patient who had sustained CCF on both teeth 31 and 41, the restorations were lost 11 times in total between the two teeth. Despite showing signs of pulp healing (i.e., dentine bridge formation, continued root development), these two teeth ended up with PN after 76 months for tooth 31 and 90 months for tooth 41.

**TABLE 2:** Distribution of treatment variables according to the pulp status at follow‐up

	Pulp healing *N* (%)	Pulp necrosis *N* (%)	*p*‐Value^a^	Odds ratio (95% CI)
Type of pulpotomy				
Partial pulpotomy (*n* = 37)	20 (54.1%)	17 (45.9%)	.15	0.420 (0.125–1.407)
Coronal pulpotomy (*n* = 19)	14 (73.7%)	5 (26.3%)		
Clinician's experience				
GDP (*n* = 15)	5 (33.3%)	10 (66.7%)	.04	
Post‐graduate (*n* = 27)	19 (70.4%)	8 (29.6%)		‐
Specialist (*n* = 14)	10 (71.4%)	4 (28.6%)		
Type of restoration				
Rebonded fragment (*n* = 11)	8 (72.7%)	3 (27.2%)	.71	
RC (*n* = 28)	17 (73.9%)	11 (39.3%)		‐
GIC (*n* = 3)	2 (66.7%)	1 (33.3%)		
RMGIC (*n* = 14)	7 (50%)	7 (50%)		
Strip crown (*n* = 27)	15 (55.6%)	12 (44.4%)	.62	1.26 (0.37–4.23)

Abbreviations: GDP, general dental practitioner; GIC, glass ionomer cement; RC, resin composite; RMGIC, resin‐modified glass ionomer cement.

^a^ Pearson Chi‐square test.

**TABLE 3:** Distribution of post‐operative variables according to pulp status at follow‐up

	Pulp healing *N* (%)	Pulp necrosis *N* (%)	*p*‐Value^a^	Odds ratio (95% CI)
Discolouration (*n* = 13)	6 (46.2%)	7 (53.8%)	.22	0.459 (0.131–1.615)
Thermal sensitivity (*n* = 19)	10 (52.6%)	9 (47.4%)	.38	0.602 (0.195–1.855)
Pain (*n* = 19)	6 (31.6%)	13 (68.4%)	<.01	0.148 (0.044–0.505)
Repeat injury (*n* = 14)	8 (57.1%)	6 (42.9%)	.75	0.821 (0.240–2.802)
Lost filling (*n* = 24)	13 (54.2%)	11 (45.8%)	.39	0.619 (0.209–1.832)
Dentine bridge (*n* = 30)	23 (76.7%)	7 (23.3%)	<.01	10.68 (2.634–43.48)

^a^ Pearson Chi‐square test.

Four teeth in the study sustained a second injury that resulted in a repeated pulp exposure, and a clinical decision was made to prophylactically remove the pulps as the roots of these teeth were fully developed. These teeth were excluded from the calculation of outcomes and the statistical analysis. Of the 56 teeth, pulp healing was observed in 34 teeth (60.7%) and 22 were deemed to have PN with infection (39.3%). The average follow‐up period for all teeth was 25.4 months. The minimum follow‐up period was 10.1 months, and the longest was 59.9 months. PN was diagnosed between 0.3 and 90.7 months, with a mean of 21.5 months.

## 4 DISCUSSION

The demographics of the study population resembled those reported in the published literature. The maxillary central incisors were the most frequently injured teeth. The frequency of CCF was almost two times higher for males compared with females. One meta‐analysis found that males were more likely to experience TDIs than females.^18^ Falls, contact sports, automobile accidents and foreign bodies striking the teeth were commonly reported causes of injury in the literature, with many TDIs occurring at home.^2^ These patterns were also reflected in this study.

When patients presented with the fractured tooth fragment, where possible, rebonding of the fragment was preferred. Of the 11 rebonded fragments, five were lost between 2 and 51 months and they were subsequently replaced with full coverage resin composite restorations using strip crowns. Reattachment of tooth fragments provides a conservative, aesthetic and cost‐effective restorative option. Furthermore, reattachment of the fragment does not affect pulp outcomes in the management of CCF.^19^ Andreasen et al.^20^ reported retention rates of 20% and 25% after 7.5 and 10.5 years, respectively, for rebonded fragments. There is a lack of consistency in the literature regarding the technique used to reattach the fragment. However, simple reattachment without tooth preparation is less technique sensitive and is the preferred technique when there is complete fragment adaptation.^21^

Compared with a RC build‐up in the same patient, the mean survival time (MST) for bonded fragments was 784 ± 528 days (25.8 ± 17.4 months), while the MST of the resin build‐ups was 701 ± 553 days (23.0 ± 18.2 months). RC was the most frequently used restorative material. Strip crowns were frequently used to assist with the restoration of the fractured tooth (46.7%). Literature pertaining to the use of strip crowns for the restoration of permanent teeth is scarce. The reported survival rate of permanent teeth restored with strip crowns in one study was 95.2% over 2 years and dropped to 88.9% after 10 years.^22^

In this study, the overall success rate of pulpotomy was lower than previous studies.^5^, ^7^, ^23^, ^24^ This study used Ca(OH)_2_ as the pulp capping agent which was consistent with previous studies. Ca(OH)_2_ has traditionally been used as a medicament for pulpotomy treatment of traumatically injured teeth with excellent success rates. Considering the popularity of calcium‐based silicate cements (CBSC), it could be suggested that an improved outcome may have been achieved with an alternate material. However, while recent literature report successful outcomes with CBSC for pulpotomies (particularly in the context of pulp therapy for carious pulp exposures), there is insufficient evidence to suggest that they are superior in the management of traumatically exposed pulps.^25^ Specific to traumatic pulp exposures, the success rates of Ca(OH)_2_ range between 87.5% and 100%,^5^, ^7^, ^8^ while the reported success rates of CBSC range between 80% and 99%.^9^, ^11^, ^12^, ^13^

Pulp necrosis was seen in the first 12 months for 60% of the cases. For most forms of TDIs in children, PN has been reported to occur within 6 months of the injury.^6^ Cvek^23^ found that all cases of PN after partial pulpotomy could be diagnosed within 26 months following treatment and he proposed the need for a 3‐year follow‐up period. Similarly, Bissinger et al.^19^ reported that the mean time interval for PN was 280.9 days (9.2 months), and most cases of PN occurred within the first 2 years after the injury. The IADT recommends clinical and radiographic evaluations after 6–8 weeks, 3, 6 months and 1 year.^1^ However, the results from this study and earlier studies suggest that a longer follow‐up period is required.

The duration of clinical experience and the radiographic presence of a dentine bridge were the only two variables significantly associated with pulp healing. Compared with GDPs, treatment provided by post‐graduate paediatric registrars and specialists had significantly better outcomes. This may be due to the limited experience of GDPs in managing dental trauma in the paediatric population.^26^ Additionally, specialists and registrars are more likely to use dental dam,^27^, ^28^ which may have also contributed to a higher success rates. The reason for not using a dental dam was not recorded for all patients in this study. This may have been attributed to poor record keeping. Alternatively, possible reasons for not using dental dams may include poor patient acceptance, time required, equipment and material cost, insufficient training and difficulty in use.^29^ Treatment in children is often challenging due to behaviour management problems and can present as a stressful experience for a GDP.^30^ Dental traumatology and behavioural management are both core components of speciality training in Paediatric Dentistry. Understandably, registrars and specialists were expected to be more knowledgeable and experienced with managing CCF than GDPs. This may translate to a more accurate assessment of the pulp status and improved execution of the procedure.

The presence of a detectable dentine bridge on radiographs was significantly associated with improved pulp prognosis (*p* < .02) in this study. Rao et al.^11^ also found dentine bridge to be more frequently observed in successful pulpotomy cases with Ca(OH)_2_. In their study, a radiographically detectable dentine bridge was seen in 90.3% of successful cases, compared with 85.7% of teeth with PN. However, the formation of a continuous dentine bridge does not preclude PN.^2^ Despite having radiographic evidence of a dentine bridge, PN was observed in 23.3% of teeth.

Other variables including age, the presence of a concomitant injury such as a luxation injury, presence of a soft tissue injury, the type of fracture, the time interval between injury and treatment and the type of pulpotomy did not have significant associations with pulp healing. The presence of a concomitant injury has been reported to be the single determinant that affects the outcome of pulpotomies.^12^ Teeth with combined injuries were reported to be 1.5 times more likely to encounter complications than teeth with fractures alone.^15^ Similarly, Robertson et al.^31^ found that the presence of a luxation injury resulted in a higher frequency of PN as damage to the PDL increased the likelihood of PN from 0% to 14%. However, it was not the case in this study. Teeth with an accompanying soft tissue injury had a higher rate of pulp healing compared with those without. Although this was not statistically significant, it was an interesting finding that has not been reported in previous studies. It was hypothesised that the direct force of the injury might have been absorbed by the soft tissue, thereby reducing the impact to the tooth. Another theory was that the patients with soft tissue injuries were more likely to seek emergency treatment sooner. However, this was unlikely, as the time interval between injury and treatment did not influence the pulp outcome. Partial pulpotomy is reported to have a more favourable prognosis compared with coronal pulpotomy.^3^ While partial pulpotomies were performed more frequently than coronal pulpotomies in this study, coronal pulpotomies had a higher percentage of favourable outcomes (*p* = .23), which was similar to the findings reported by an earlier study.^9^

Having no pain during the follow‐up period (adjusted OR = 10.4 95% CI 2.27–47.59) and the stage of root development (adjusted OR = 0.17 95% CI 0.03–0.91) were associated with pulp healing. Both were identified as significant predictors of outcome. The association between the stage of root development and pulp healing is often disputed in the literature. In this study, immature teeth had a better prognosis compared with mature teeth. Similar findings have been reported in other studies. Wang et al.^9^ also observed that teeth with immature tooth development had a better prognosis than those with mature roots. However, Robertson et al.^31^ reported that the stage of root development was only critical in determining the risk of PN after a crown fracture in the presence of a concomitant luxation injury. Conversely, earlier studies found similar success rates for teeth with both immature and mature roots, and they concluded that the stage of root development did not affect the treatment outcome.^5^, ^7^ In terms of pain history, pulp healing was more likely to occur in teeth where the patient did not report pain at the follow‐up appointments. Of the 19 teeth with a history of pain, PN was seen in 13 (68.4%). The pain resolved in the remaining six teeth (31.6%). Further investigation will be required to identify the cause of the pain and the factors which contributed to its resolution.

One of the major limitations of this study was the lack of standardised clinical records. The missing data meant that the sample size was small and statistical analysis could not be performed for all variables. Another limitation was that many teeth were excluded due to insufficient follow‐up. In many instances, the patients were discharged back to their local dentist or to their local oral health clinics. Patients that had subsequent injuries or had reported symptoms were more likely to be followed up at WCOH. This would have likely skewed the study population towards a poorer outcome and may explain the lower success rate of pulpotomy in this study when compared with those reported in the literature. While pulpotomy remains a viable treatment option, appropriate case selection and further training in the use of dental dam in the hospital setting may be required to ensure improved pulp healing outcomes. The development of clinical protocols for trauma scenarios in line with evidence‐based procedures would also be advantageous for better outcomes. Furthermore, a longer follow‐up period should be considered to monitor for late‐stage complications.

## AUTHOR CONTRIBUTIONS

None.

## ACKNOWLEDGEMENT

Open access publishing facilitated by The University of Sydney, as part of the Wiley ‐ The University of Sydney agreement via the Council of Australian University Librarians.

## CONFLICT OF INTEREST

The authors confirm that they have no conflict of interest.

## Data Availability

Data sharing is not applicable to this article as no new data were created or analyzed in this study.
